# A novel pathogenic frameshift variant unmasked by a large de novo deletion at 13q21.33-q31.1 in a Chinese patient with neuronal ceroid lipofuscinosis type 5

**DOI:** 10.1186/s12881-020-01039-5

**Published:** 2020-05-11

**Authors:** Wei Li, Xin Fan, Yue Zhang, Limei Huang, Tingting Jiang, Zailong Qin, Jiasun Su, Jingrong Luo, Shang Yi, Shujie Zhang, Yiping Shen

**Affiliations:** 1 Genetic and Metabolic Central Laboratory, Birth Defect Prevention Research Institute, Maternal and Child Health Hospital, Children’s Hospital of Guangxi Zhuang Autonomous Region, Nanning, 530002 China; 2 Department of Medical Genetics and Molecular Diagnostic Laboratory, Shanghai Children’s Medical Center, Shanghai Jiao Tong University School of Medicine, Shanghai, 200127 China; 3 Division of Genetics and Genomics, Boston Children’s Hospital, Boston, USA; 4 Department of Neurology, Harvard Medical School, Boston, MA 02115 USA

**Keywords:** Neuronal ceroid lipofuscinoses, *CLN5*, CNV, Exome sequencing

## Abstract

**Background:**

Neuronal ceroid lipofuscinosis type 5 (CLN5) is a rare form of neuronal ceroid lipofuscinoses (NCLs) which are a group of inherited neurodegenerative diseases characterized by progressive intellectual and motor deterioration, visual failure, seizures, behavioral changes and premature death. CLN5 was initially named Finnish variant late infantile NCL, it is now known to be present in other ethnic populations and with variable age of onset. Few CLN5 patients had been reported in Chinese population.

**Case presentation:**

In this paper, we report the symptoms of a Chinese patient who suffer from developmental regression and grand mal epilepsy for several years. The DNA was extracted from peripheral blood of proband and both parents, and then whole exome sequencing was performed using genomic DNA. Both sequence variants and copy number variants (CNVs) were analyzed and classified according to guidelines. As the result, a novel frameshift mutation c.718_719delAT/p.Met240fs in *CLN5* and a de novo large deletion at 13q21.33-q31.1 which unmasked the frameshift mutation were identified in the proband. Despite the large de novo deletion, which can be classified as a pathogenic copy number variant (CNV), the patient’s clinical presentation is mostly consistent with that of CLN5, except for early developmental delay which is believed due to the large deletion. Both variants were detected simultaneously by exome sequencing.

**Conclusions:**

This is the first report of whole gene deletion in combination with a novel pathogenic sequence variant in a CLN5 patient. The two mutations detected with whole exome sequencing simultaneously proved the advantage of the sequencing technology for genetic diagnostics.

## Background

NCLs are clinically and genetically heterogeneous group of neurodegenerative lysosomal storage disorders characterized by the intracellular accumulation of autofluorescent lipopigment [1, 2], presented with progressive intellectual and motor deterioration, visual failure, seizures, behavioral changes and premature death [2–4], mostly with autosomal recessive mode of inheritance [5]. The old classification of NCLs was based on the age of onset of the symptoms, which consisted of four major groups i.e. infantile, late infantile, juvenile and adult. The new classification of NCLs is based on causative genes. Currently a total of 13 genes had been identified as the monogenic causes of NCLs, including CLN1/PPT1(OMIM #256730), CLN2/TPP1(OMIM #204500), CLN3(OMIM #204200), CLN4/DNAJC5(OMIM #204300), CLN5(OMIM #256731), CLN6(OMIM #601780), CLN7/MFSD8(OMIM #610951), CLN8(OMIM #600143), CLN10/CTSD (OMIM #610127), CLN11/GRN (OMIM #614706), CLN12/ATP13A2(OMIM #610513), CLN13/CTSF (OMIM #615362), CLN14/KCTD7 (OMIM #611725) [3]. Juvenile neuronal ceroid lipofuscinoses, previously described as CLN9 variant, are reclassified as CLN5 disease [6].

CLN5 was believed to be a rare form of NCL with region specific distribution. It was initially identified in a region of Finland. The patients usually exhibited symptoms at the age of 4–7 years, thus it was named as Finnish variant late infantile neuronal ceroid lipofuscinosis (vLINCL) [4, 7]. The identification of causative *CLN5* gene prompted the screening of CLN5 patients from other regions [8]. Pineda-Trujillo et al. reported the first case of CLN5 patient outside northern Europe with atypical late onset [9]. Xin et al. reported additional CLN5 cases from a diverse population and variable age of onset [10]. Evidence indicated NCL patients with *CLN5* mutations are more common than initially believed and can be found in many other ethnic populations. One of the patient reported by Xin et al. was Chinese. Another Chinese CLN5 patient was recently identified [11]. We believe *CLN5* associated NCL patients are under reported in China due to a lack of clinical expertise and routine molecular testing for them.

Here we report a Chinese patient with a novel pathogenic variant c.718_719delAT/p.Met240fs which is unmasked by a de novo 7.1 Mb 13q21.33-q31.1 deletion. This is the first report that whole CLN5 gene deletion occurred in CLN5 patient. Exome sequencing enabled the simultaneous detection of the point mutation and CNV.

## Case presentation

The patient was a boy, born at 37 weeks of gestation by a G1P1 healthy woman. The delivery was uneventful. The birth weight was 3.450 kg (~ + 0.5 SD). Both parents were healthy and unrelated. There are no family history of developmental delay or regression.

**Fig. 1:**
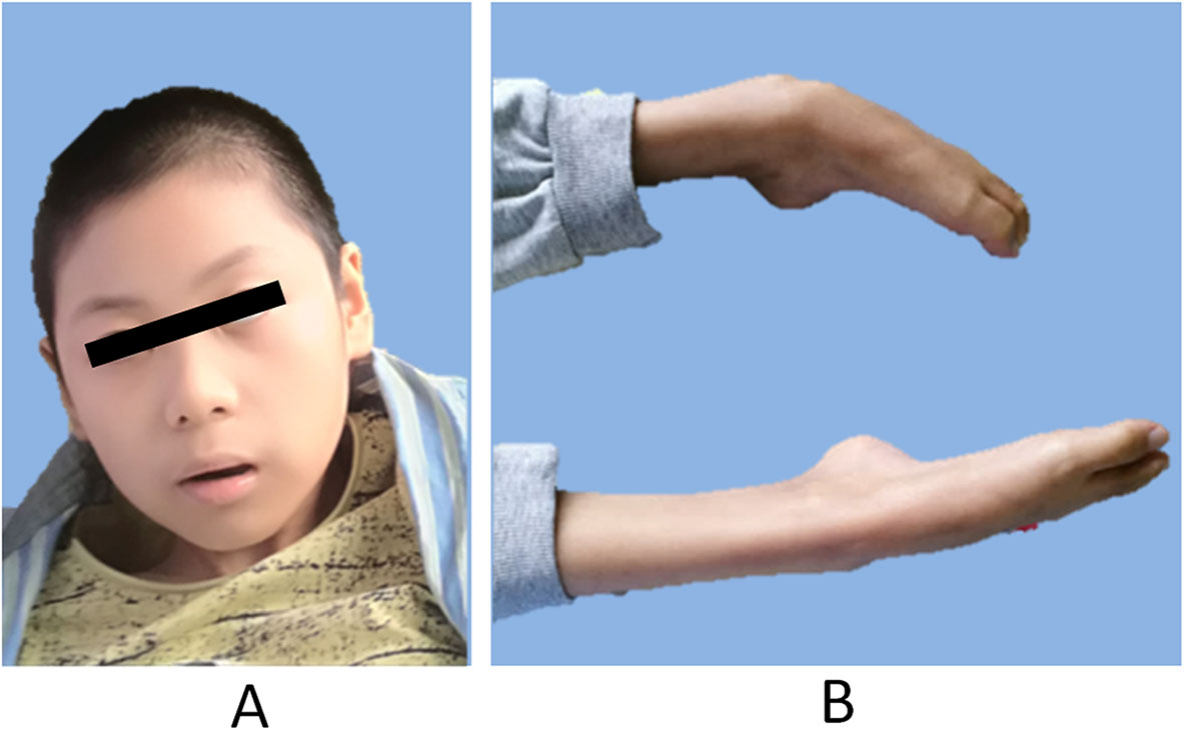
Patient with strabismus and gazing fixation (**a**); spastic rigid legs (**b**)

The proband had early developmental delay. Hypotonia was reported. He could hold his head at 3 to 4 months of age and sit by himself at 6 to 7 months of age. At the age of one, he started walking with poor stability as well as began talking with poor articulation. He could recite simple poems when he was about 3 to 4 years old. The patient exhibited regression after age 4, initially with language difficulty which gradually lead to a total loss of speaking capability after 7 years of age. The proband was examined at the hospital when he was 5 years old for poor communication capability. His cognitive competence was below average. He could speak 3 to 4 words and his audio-visual sensitivity was intact. Although he could say numbers under 30, he could not sort them by order. Through various rehabilitation treatments such as occupational therapy, language training, transcranial magnetic stimulation, conductive education and sensory integration training, his fine motor and balanced skills were poor yet his cognitive competence slightly improved. His pronunciation was clearer than before. But he gradually lost his ability to walk between 4 to 7 years of age and is now completely incapable of standing and stepping. He also experienced severe convulsions (grand mal epilepsy) started at the age of 4 and it had been continuing since. Each episode of convulsion lasted 1–2 min and recurred at 1 to 2 month interval. His vision started to decline since the age of 6 and now is completely blind at the age of 10. Other symptoms included dystaxia, nystagmus, visual fixation, rigidity of limbs with spastic appearance (Fig. 1a and b). Currently, he has no mental capacity, no response to name calling. He could only intake liquid food because of hypotonia. The brain MRI performed at the age of 5 showed brain atrophy involving mesencephalon, pons and cerebellum.

The peripheral blood samples were collected from the proband and his parents after obtaining informed consent. DNA extracted from peripheral blood samples were subjected for whole exome sequencing using commercial kits. We performed target capture with Illumina TruSeq kit (Illumina, Inc. USA), and did sequencing on HiSeq platform. Sequencing data were analyzed with a TAGK pipeline and the variants were further filtered and prioritized in TGex (LifeMap Sciences, Ltd.Israel). CNV using whole exome data were generated by a custom program using batch-controlled data as reference.

**Fig. 2:**
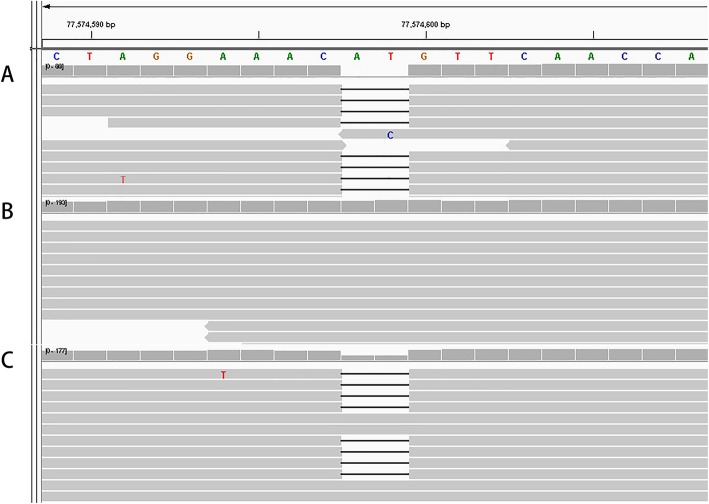
The frameshift mutation was detected in the genomes of proband (**a**) and mother’s sample (**c**) but not in father’s sample (**b**)

**Fig. 3:**
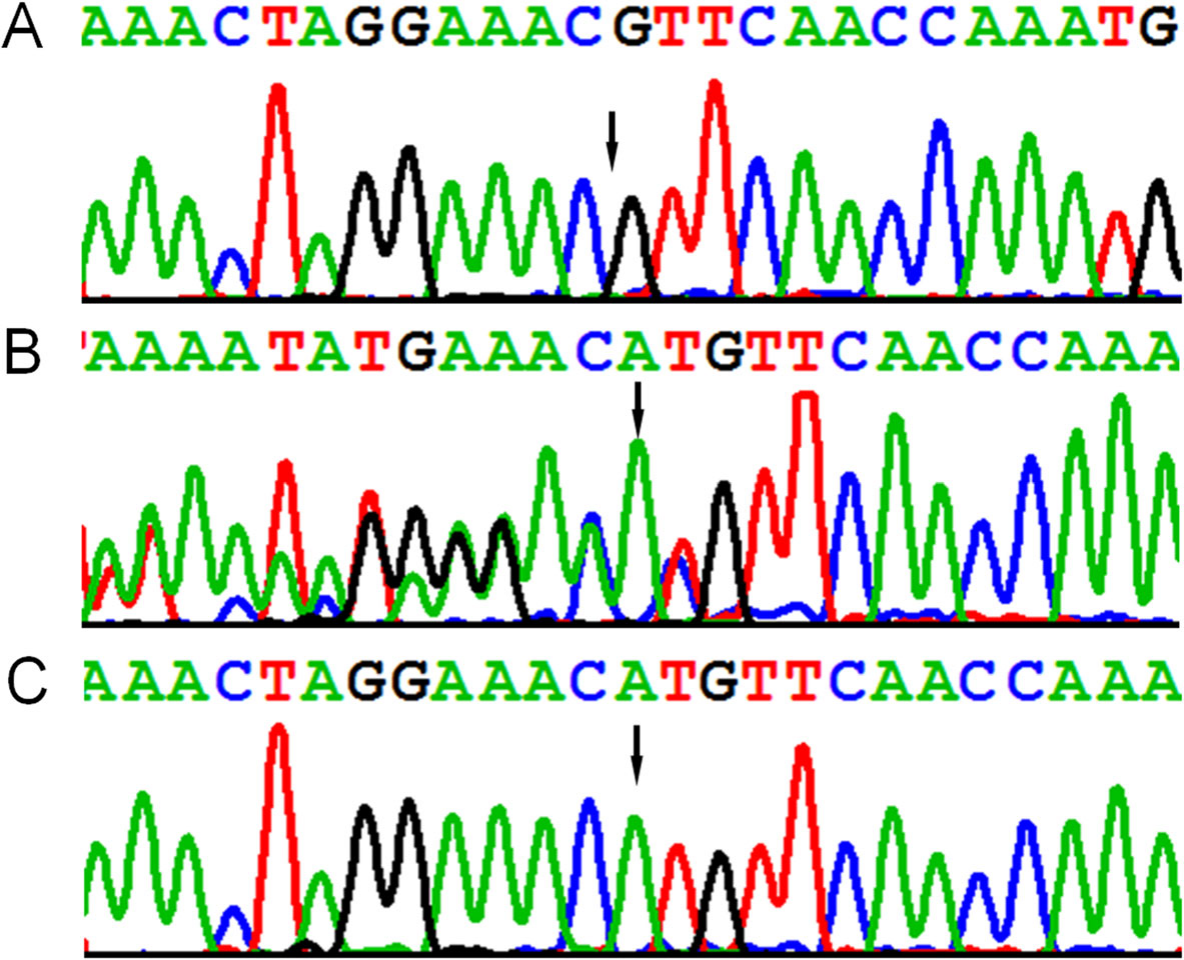
Sanger sequencing chromatograms show a frameshift variant c.718_719delAT/ p.Met240fs in the proband (**a**) and his mother (**b**), not in his father (**c**)

**Fig. 4:**
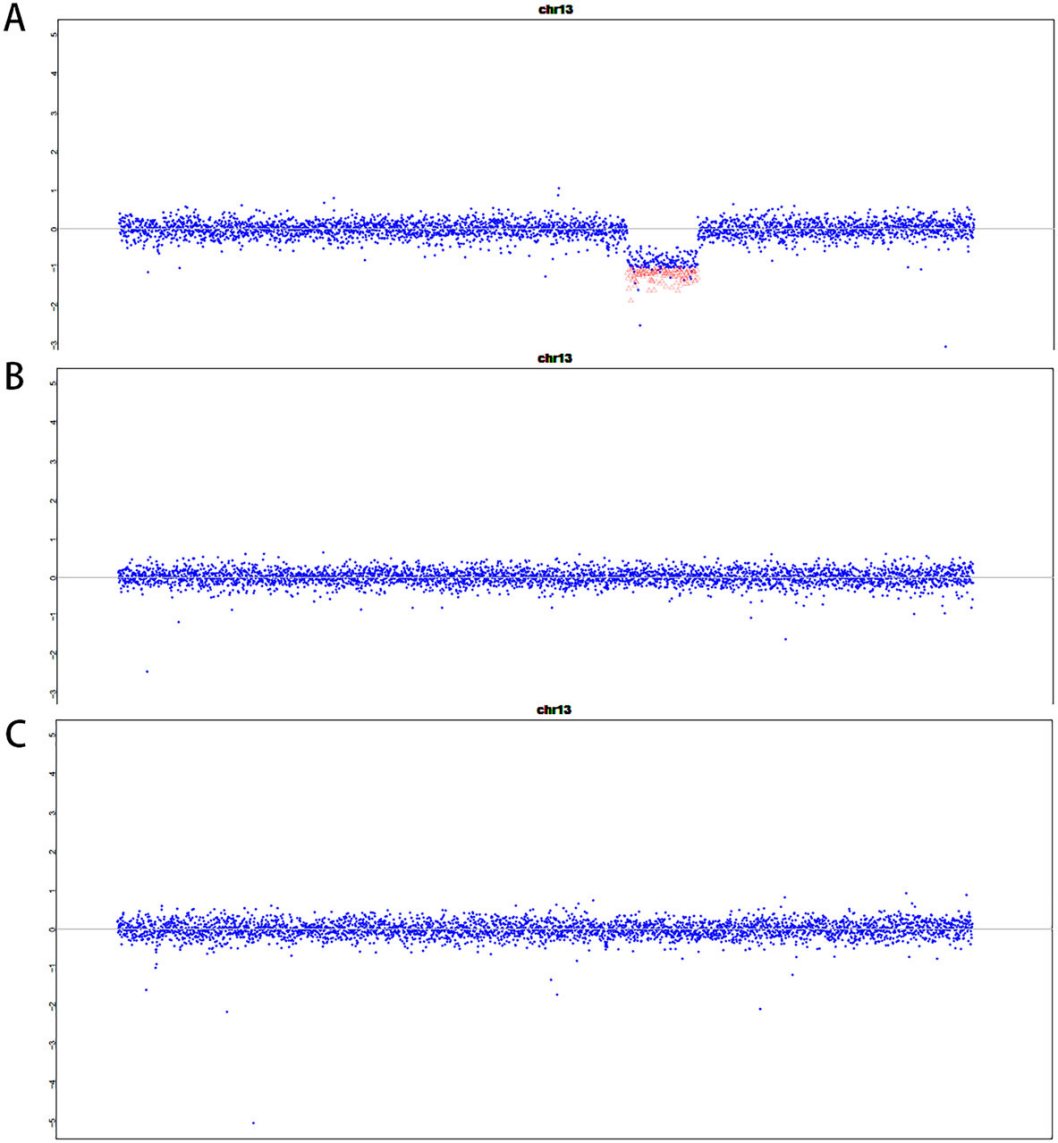
CNV analysis revealed a heterozygous deletion on chromosome 13 at 13q21.33q31.3(72,306,245–79,485,372) in proband (**a**), the deletion was absent from the paternal (**b**) and maternal (**c**) chromosomes

As the result, exome sequencing data analysis revealed an apparent homozygous frameshift variant c.718_719delAT/p.Met240fs in *CLN5* gene. This frameshift variant was not previously reported and can be classified as a pathogenic variant according to the ACGM/AMP sequence variants classification guidelines [12]. The variant met at least the following evidence of support for pathogenicity: PVS1, PM2 and PP4. Parental sample testing confirmed the maternal origin of the frameshift variant (Fig. 2). The paternal sample did not carry this frameshift mutation. The result of exome sequencing was confirmed by Sanger sequencing (Fig. 3). The frameshift variant was inherited from his mother. We detected a deletion encompassing the whole CLN5 gene in proband by CNV analysis, and the deletion was absent from parents (Fig. 4). This deletion was de novo and large, containing more than 70 Refseq genes including a haploinsufficient gene *EDNRB* (OMIM 131244). We listed Refseq genes and their putative function in an additional table (Additional file 1: Table S1). This deletion can be classified as pathogenic and is described as arr. 13q21.33q31.3 (72,306,245–79,485,372) × 1dn. Thus the homozygous frameshift mutation is actually a hemizygous mutation which is unmasked by the heterozygous deletion.

## Discussion and conclusions

The majority of *CLN5* pathogenic and likely pathogenic variants in ClinVar (https://www.ncbi.nlm.nih.gov/clinvar/?term=CLN5%5Bgene%5D, accessed Dec.12018) are null variants including 31 frameshift, 15 nonsense, 3 splicing and 3 intragenic deletion variants. In addition, there are 11 pathogenic missense variants. The frameshift variant detected in our patient had not been reported in HGMD or ClinVar. Large deletion CNVs including *CLN5* and of variable size and boundary including monosomy 13q had been reported in over 20 individuals in Clinvar, but none was detected in CLN5 patients. Our patient is the first of such situation that a large deletion involving *CLN5* is compounded with a pathogenic frameshift variant in *CLN5* gene.

Children with CLN5 disease often have normal early development before regression initiates around the age of four. Our patient exhibited early developmental delay involving motor function and language usage. It is conceivable that the early developmental delay is due to the large de novo deletion since overlapping deletions in postnatal patients reported in Decipher (for example 299003, 261869, 1583 and 250039) often exhibited global developmental delay and hypotonia. This large deletion involved *EDNRB* gene which is associated with Waardenburg syndrome type 4A (WS4A) and susceptibility to Hirschsprung disease. Both conditions exhibit incomplete penetrance and the penetrance is dose dependent. Biallelic mutations lead to high penetrance. Our patient with one copy deletion did not exhibit features of WS4A or Hirschsprung disease is consistent with the reduce penetrance of these conditions.

The clinical features of Finnish patients with CLN5 mutations have been well described in 1980’s [13, 14]. Visual deterioration, decreased attention span, gait ataxia, tremors, seizure, motor difficulty and cognitive regression were reported at early stages of CLN5 in many clinical cases [9, 10, 15–17]. Holmberg et al. reported the clumsiness and concentration disturbances between the age of 2 to 6, followed by mental decline and visual deterioration in eight Finnish vLINCL patients [18, 19]. Epileptic seizures, myoclonia, and ataxia started between the age of 7 to 11, which are the major reasons for severe disability. Simonati et al. characterized the phenotypic profile of 15 children with CLN5 disease [20]. The onset of the disease for these patients ranged from 2 years old to 7 years and 6 months old (median 5y). The most common symptoms included impaired learning and cognition, hyperactivity, aggression, intolerance and motor stereotypes. Seizures and vision impairment occurred relatively later, usually 3 to 4 years after onset of the disease.

We reviewed all previously published CLN5 cases and plotted the age of onset distributions for key features (Additional file 2: Figure S1). Most patients have vision problems around 5–10 years of age. In some patients, visual failure is the first presenting feature. Vision decline would continue and possibly lead to blindness 1 to 5 years after the onset of the symptom [9, 21]. The visual impairment of our patient happened at age 6 (Additional file 2: Figure S1 a). The penetrance of visual impairment reached 80% based on the published cases. This feature has the highest penetrance among other CLN5-related features.

Motor clumsiness mainly occurs around 4 years to 8 years old (Additional file 2: Figure S1 b). Motor impairment or regression, fine motor and gross motor problems, progressive motor retardation, and total loss of motor functions would follow after the initial onset of the symptom [10, 15, 17, 22]. Loss of the ability to walk independently occurs around age 8 to age 13 (Additional file 2: Figure S1 c). In some patients, it may take more than 10 years for motor skill to deteriorate from unsteady gait to inability to walk [23]. Our patient gradually lost motor skills from 4 years old to 7 years old and became bedridden 6 years after the symptom onset. The penetrance of motor clumsinessis 71%. The penetrance of total loss of walking capability is as low as 19% based on the current published cases however the penetrance is age dependent.

Intellectual disability occurs mainly at age 3 to 7 which affects preschool and school age children (Additional file 2: Figure S1 d). The penetrance of intellectual disability is 79%. The onset of language impairment ranged from 2 to 11 years of age (Additional file 2: Figure S1 e). The penetrance of language regression is 60%. Our patient presented language impairment at the age of 4 and showed below the average cognitive competence at the age of 5.

The onset of epilepsy among CLN5 patients mainly occurs around 4–10 years of age and epilepsy penetrance is as high as 66% (Additional file 2: Figure S1 f). Motor impairment usually occurs several years after the onset of seizure [15, 18] and ataxia occurs at 4 to 11 years of age in most patients (Additional file 2: Figure S1 g) with a penetrance of 26% based on reported cases. Both epilepsy and dystaxia appeared when our patient was 4 years old. The initial symptoms of our patient started at age of 4 including decline of language capacity, difficulty in walking and convulsion. It is rarely reported that these three features presented at the initial stage of disease onset.

In this study, we reported the first case of CLN5 patient with a large de novo heterozygous deletion which unmasked a novel pathogenic frameshift variant. Figure S1 presented that most of the CLN5-related features in our patient has an earlier onset in comparison to the average age of symptoms onset, it may well be attributable to the presence of a large deletion accompanying with a loss of function variant. The simultaneous detection of both large and small pathogenic variants in CLN5 demonstrated the advantage of exome sequencing. The molecular findings and the phenotypic information of this patient can help to delineate genotype-phenotype correlations for CLN5.

## Supplementary information

**Additional file 1 **: **Table S1**. The 74 Refseq genes in the deleted region of chromosome 13. The table includes gene name, OMIM number and their putative function. Some are pseudogenes or non-coding RNA without known functions.

**Additional file 2 **: **Figure S1**. The age of onset of CLN5-related features based on published cases. These symptoms include visual failure, motor impairment, walking ability loss, intellectual disability, language impairment, seizure, ataxia. The X-axis shows the age at symptom onset. Red arrows indicate the age of onset of our patient for the respective features.

## Data Availability

Clinical data and sequencing data in this study were provided by corresponding author upon request following approval of the ethics committee of Maternal and Child Health Hospital of Guangxi Zhuang Autonomous Region. The data used and analyzed in the present report were deposited in the Sequence Read Archive (SRA) database. The data are accessible via the SRA accession: PRJNA628102; or via the links: https://dataview.ncbi.nlm.nih.gov/object/PRJNA628102; https://trace.ncbi.nlm.nih.gov/Traces/sra/?run=SRR11609212; https://trace.ncbi.nlm.nih.gov/Traces/sra/?run=SRR11609213;https://trace.ncbi.nlm.nih.gov/Traces/sra/?run=SRR11609214.

## Abbreviations

CLN5: Neuronal ceroid lipofuscinosis type 5
NCLs: Neuronal ceroid lipofuscinoses
CNVs: Copy number variants
vLINCL: variant late infantile neuronal ceroid lipofuscinosis
MRI: Magnetic Resonance Imaging
