# Supplementary material for: A novel pathogenic frameshift variant unmasked by a large de novo deletion at 13q21.33-q31.1 in a Chinese patient with neuronal ceroid lipofuscinosis type 5
Source: BMC Med Genet. 2020 May 11;21:100. doi: 10.1186/s12881-020-01039-5 (PMC7216669; doi:10.1186/s12881-020-01039-5)
Supplement: Supplementary file 1 — Additional file 1 : Table S1. The 74 Refseq genes in the deleted region of chromosome 13. The table includes gene name, OMIM number and their putative function. Some are pseudogenes or non-coding RNA without known functions. [file 12881_2020_1039_MOESM1_ESM.docx]

**Additional file 1**

**Table S1.** **The 74 Refseq genes in the deleted region of chromosome 13**

| **Gene Name** | **OMIM** | | | **Putative Function** |  |
| --- | --- | --- | --- | --- | --- |
| *DACH1* | 603803 | | | Transcription factor that is involved in regulation of organogenesis[1]. |  |
| *MZT1* | 613448 | | | - Required for gamma-tubulin complex recruitment to the centrosome[2]. |  |
| *BORA* | 610510 | | | - Required for the activation of AURKA at the onset of mitosis[[3](#_ENREF_15)]. |  |
| *DIS3* | 607533 | | Putative catalytic component of the RNA exosome complex which has 3'-5' exoribonuclease activity and participates in a multitude of cellular RNA processing and degradation events[4,5]. | | |
| *PIBF1* | 607532 | | Plays a role in ciliogenesis[6]. | | |
| *KLF5* | 602903 | - Transcription factor that binds to GC box promoter elements. Activates the transcription of these genes[7]. | | |  |
| *KLF12* | 607531 | - KLF12 confers strong transcriptional repression to the AP-2-alpha gene and is reciprocally regulated by AP-2-alpha.Binds to a regulatory element (A32) in the AP-2-alpha gene promoter[8]. | | |  |
| *TBC1D4* | 612465 | - May act as a GTPase-activating protein for RAB2A, RAB8A, RAB10 and RAB14. Isoform 2 promotes insulin-induced glucose transporter SLC2A4/GLUT4 translocation at the plasma membrane, thus increasing glucose uptake[9,10]. | | |  |
| *COMMD6* | 612377 | May modulate activity of cullin-RING E3 ubiquitin ligase (CRL) complexes [11]. Down-regulates activation of NF-kappa-B. Inhibits TNF-induced NFKB1 activation[12,13]. | | |  |
| *UCHL3* | 603090 | - A member of deubiquitinating enzyme (DUB). DUB that controls levels of cellular ubiquitin through processing of ubiquitin precursors and ubiquitinated proteins.Indirectly increases the phosphorylation of IGFIR, AKT and FOXO1 and promotes insulin-signaling and insulin-induced adipogenesis. Required for stress-response retinal, skeletal muscle and germ cell maintenance. May be involved in working memory[14-17]. | | |  |
| *LMO7* | 604362 | - LMO7 positively regulates many Emery-Dreifuss muscular dystrophy relevant genes (including emerin), and is feedback-regulated by binding to emerin[18]. | | |  |
| *KCTD12* | 610521 | - Auxiliary subunit of GABA-B receptors that determine the pharmacology and kinetics of the receptor response. Increases agonist potency and markedly alter the G-protein signaling of the receptors by accelerating onset and promoting desensitization[19]. | | |  |
| *CLN5* | 608102 | Plays a role in influencing the retrograde trafficking of lysosomal sorting receptors SORT1 and IGF2R from the endosomes to the trans-Golgi network by controlling the recruitment of retromer complex to the endosomal membrane. Regulates the localization and activation of RAB7A which is required to recruit the retromer complex to the endosomal membrane[[20](#_ENREF_32)]. | | |  |
| *FBXL3* | 605653 | FBXL3 is involved in the pathway protein ubiquitination, which is part of protein modification.Substrate-recognition component of the FBXL3 E3 ubiquitin ligase complex involved in circadian rhythm function. Plays a key role in the maintenance of both the speed and the robustness of the circadian clock oscillation. The FBXL3 complex mainly acts in the nucleus and mediates ubiquitination and subsequent degradation of CRY1 and CRY2 [21-23]. | | |  |
| *MYCBP2* | 610392 | Atypical E3 ubiquitin-protein ligase which specifically mediates ubiquitination of threonine and serine residues on target proteins. It was required for the formation of major central nervous system axon tracts, and regulates axon guidance in the olfactory system. It will catalyze ubiquitination of threonine and/or serine residues on NMNAT2, consequences of threonine and/or serine ubiquitination are however unknown [[24](#_ENREF_36)]. It mediates ubiquitination and subsequent proteasomal degradation of TSC2/tuberin[25,26]. | | |  |
|  |  |  | | |  |
| *SCEL* | 604112 | May function in the assembly or regulation of proteins in the cornified envelope. The presence of sciellin in simple epithelia suggests that the various regions of sciellin (e.g., the LIM domain) play different roles in epithelial function[27]. | | |  |
|  |  |  | | |  |
| *SLAIN1* | 610491 | Microtubule plus-end tracking protein that might be involved in the regulation of cytoplasmic microtubule dynamics, microtubule organization and microtubule elongation[28]. | | |  |
| *EDNRB* | 131244 | Non-specific receptor for endothelin 1, 2, and 3. Mediates its action by association with G proteins that activate a phosphatidylinositol-calcium second messenger system[29]. | | |  |
| *POU4F1* | 601632 | - Regulates the expression of specific genes involved in differentiation and survival within a subset of neuronal lineages. Exerts its major developmental effects in somatosensory neurons and in brainstem nuclei involved in motor control[30]. | | |  |
| *RPL35AP31* | N/A | Unknown | | |  |
| *RPS10P21* | N/A | Unknown | | |  |
| *RPL18AP17* | N/A | Unknown | | |  |
| *RPL21P110* | N/A | Unknown | | |  |
| *RNU6-80P* | N/A | Unknown | | |  |
| *RNU6-79P* | N/A | Unknown | | |  |
| *PSMD10P3* | N/A | Unknown | | |  |
| *FABP5P1* | N/A | Unknown | | |  |
| *RNU6-66P* | N/A | Unknown | | |  |
| *RNU7-89P* | N/A | Unknown | | |  |
| *RNU4-10P* | N/A | Unknown | | |  |
| *RNY1P8* | N/A | Unknown | | |  |
| *MARK2P12* | N/A | Unknown | | |  |
| *LINC00392* | N/A | Unknown | | |  |
| *LINC00402* | N/A | It is correlated with reduced survival time in patients with metastatic melanoma[31]. | | |  |
| *RNY1P5* | N/A | Unknown | | |  |
| *RPL21P108* | N/A | Unknown | | |  |
| *LOC100288208* | N/A | Unknown | | |  |
| *LINC00381* | N/A | Unknown | | |  |
| *LINC00347* | N/A | Unknown | | |  |
| *RIOK3P1* | N/A | Unknown | | |  |
| *RNU6-38P* | N/A | Unknown | | |  |
| *SSR1P2* | N/A | Unknown | | |  |
| *CTAGE11P* | N/A | Unknown | | |  |
| *LINC01078* | N/A | Unknown | | |  |
| *LMO7-AS1* | N/A | Unknown | | |  |
| *FAM204CP* | N/A | Unknown | | |  |
| *LMO7DN* | N/A | Unknown | | |  |
| *LMO7DN-IT1* | N/A | Unknown | | |  |
| *RN7SL571P* | N/A | Unknown | | |  |
| *ACOD1* | N/A | *cis*-Aconitate decarboxylase (CAD, also known as ACOD1 or Irg1) converts *cis*-aconitate to itaconate and plays central roles in linking innate immunity with metabolism and in the biotechnological production of itaconic acid by *Aspergillus terreus*[32]. | | |  |
| *RPL7P44* | N/A | Unknown | | |  |
| *DHX9P1* | N/A | Unknown | | |  |
| *MYCBP2-AS1* | N/A | Unknown | | |  |
| *SCEL-AS1* | N/A | Unknown | | |  |
| *RNY3P7* | N/A | Unknown | | |  |
| *SPTLC1P5* | N/A | Unknown | | |  |
| *LOC100129307* | N/A | Unknown | | |  |
| *MIR3665* | N/A | [Expression](javascript:;) is upregulation in HIV-associated neurocognitive disorder cases compared with the non-HAND group (P < 0.05) [33]. | | |  |
| *EDNRB-AS1* | N/A | Unknown | | |  |
| *RN7SL810P* | N/A | Unknown | | |  |
| *LINC01069* | N/A | Unknown | | |  |
| *LINC00446* | N/A | Unknown | | |  |
| *RNF219-AS1* | N/A | Unknown | | |  |
| *SRGNP1* | N/A | Unknown | | |  |
| *RNY3P3* | N/A | Unknown | | |  |
| *RPL31P54* | N/A | Unknown | | |  |
| *ELOCP23* | N/A | Unknown | | |  |
| *RNF219* | N/A | In Alzheimer's disease(AD) patients, the  *RNF219*  variants were found to be associated with increased anxiety levels. There is data indicates a novel synergistic activity *APOE* and *RNF219* in the modulation of behavioral traits of female mild cognitive impairment and AD patients[34]. | | |  |
| *RPL21P111* | N/A | Unknown | | |  |
| *LINC00331* | N/A | Unknown | | |  |
| *HSPD1P8* | N/A | Unknown | | |  |
| *CCT5P2* | N/A | Unknown | | |  |
| *CCT5-2P* | N/A | Unknown | | |  |
| *BTF3P11* | N/A | Unknown | | |  |

# References

1. Wu K, Yang Y, Wang C, Davoli MA, D'Amico M, Li A *et al*: DACH1 inhibits transforming growth factor-beta signaling through binding Smad4. J Biol Chem. 2003; 278(51):51673-84.

2. Hutchins JR, Toyoda Y, Hegemann B, Poser I, Heriche JK, Sykora MM *et al*: Systematic analysis of human protein complexes identifies chromosome segregation proteins. Science. 2010; 328(5978):593-9.

3. Hutterer A, Berdnik D, Wirtz-Peitz F, Zigman M, Schleiffer A, Knoblich JA: Mitotic activation of the kinase Aurora-A requires its binding partner Bora. Dev Cell. 2006; 11(2):147-57.

4. Preker P, Nielsen J, Kammler S, Lykke-Andersen S, Christensen MS, Mapendano CK *et al*: RNA exosome depletion reveals transcription upstream of active human promoters. Science. 2008; 322(5909):1851-4.

5. Tomecki R, Kristiansen MS, Lykke-Andersen S, Chlebowski A, Larsen KM, Szczesny RJ *et al*: The human core exosome interacts with differentially localized processive RNases: hDIS3 and hDIS3L. EMBO J. 2010; 29(14):2342-57.

6. Wheway G, Schmidts M, Mans DA, Szymanska K, Nguyen TT, Racher H *et al*: An siRNA-based functional genomics screen for the identification of regulators of ciliogenesis and ciliopathy genes. Nat Cell Biol. 2015; 17(8):1074-87.

7. Sogawa K, Imataka H, Yamasaki Y, Kusume H, Abe H, Fujii-Kuriyama Y: cDNA cloning and transcriptional properties of a novel GC box-binding protein, BTEB2. Nucleic Acids Res. 1993; 21(7):1527-32.

8. Roth C, Schuierer M, Gunther K, Buettner R: Genomic structure and DNA binding properties of the human zinc finger transcriptional repressor AP-2rep (KLF12). Genomics. 2000; 63(3):384-90.

9. Baus D, Heermeier K, De Hoop M, Metz-Weidmann C, Gassenhuber J, Dittrich W *et al*: Identification of a novel AS160 splice variant that regulates GLUT4 translocation and glucose-uptake in rat muscle cells. Cell Signal. 2008; 20(12):2237-46.

10. Miinea CP, Sano H, Kane S, Sano E, Fukuda M, Peranen J *et al*: AS160, the Akt substrate regulating GLUT4 translocation, has a functional Rab GTPase-activating protein domain. Biochem J. 2005; 391(Pt 1):87-93.

11. Mao X, Gluck N, Chen B, Starokadomskyy P, Li H, Maine GN *et al*: COMMD1 (copper metabolism MURR1 domain-containing protein 1) regulates Cullin RING ligases by preventing CAND1 (Cullin-associated Nedd8-dissociated protein 1) binding. J Biol Chem. 2011; 286(37):32355-65.

12. Burstein E, Hoberg JE, Wilkinson AS, Rumble JM, Csomos RA, Komarck CM *et al*: COMMD proteins, a novel family of structural and functional homologs of MURR1. J Biol Chem. 2005; 280(23):22222-32.

13. de Bie P, van de Sluis B, Burstein E, Duran KJ, Berger R, Duckett CS *et al*: Characterization of COMMD protein-protein interactions in NF-kappaB signalling. Biochem J. 2006; 398(1):63-71.

14. Wada H, Kito K, Caskey LS, Yeh ET, Kamitani T: Cleavage of the C-terminus of NEDD8 by UCH-L3. Biochem Biophys Res Commun. 1998; 251(3):688-92.

15. Setsuie R, Sakurai M, Sakaguchi Y, Wada K: Ubiquitin dimers control the hydrolase activity of UCH-L3. Neurochem Int. 2009; 54(5-6):314-21.

16. Dennissen FJ, Kholod N, Hermes DJ, Kemmerling N, Steinbusch HW, Dantuma NP *et al*: Mutant ubiquitin (UBB+1) associated with neurodegenerative disorders is hydrolyzed by ubiquitin C-terminal hydrolase L3 (UCH-L3). FEBS Lett. 2011; 585(16):2568-74.

17. Iphofer A, Kummer A, Nimtz M, Ritter A, Arnold T, Frank R *et al*: Profiling ubiquitin linkage specificities of deubiquitinating enzymes with branched ubiquitin isopeptide probes. Chembiochem. 2012; 13(10):1416-20.

18. Holaska JM, Rais-Bahrami S, Wilson KL: Lmo7 is an emerin-binding protein that regulates the transcription of emerin and many other muscle-relevant genes. Hum Mol Genet. 2006; 15(23):3459-72.

19. Schwenk J, Metz M, Zolles G, Turecek R, Fritzius T, Bildl W *et al*: Native GABA(B) receptors are heteromultimers with a family of auxiliary subunits. Nature. 2010; 465(7295):231-5.

20. Mamo A, Jules F, Dumaresq-Doiron K, Costantino S, Lefrancois S: The role of ceroid lipofuscinosis neuronal protein 5 (CLN5) in endosomal sorting. Mol Cell Biol. 2012; 32(10):1855-66.

21. Busino L, Bassermann F, Maiolica A, Lee C, Nolan PM, Godinho SI *et al*: SCFFbxl3 controls the oscillation of the circadian clock by directing the degradation of cryptochrome proteins. Science. 2007; 316(5826):900-4.

22. Yoo SH, Mohawk JA, Siepka SM, Shan Y, Huh SK, Hong HK *et al*: Competing E3 ubiquitin ligases govern circadian periodicity by degradation of CRY in nucleus and cytoplasm. Cell. 2013; 152(5):1091-105.

23. Scott DC, Rhee DY, Duda DM, Kelsall IR, Olszewski JL, Paulo JA *et al*: Two Distinct Types of E3 Ligases Work in Unison to Regulate Substrate Ubiquitylation. Cell. 2016; 166(5):1198-214 e24.

24. Pao KC, Wood NT, Knebel A, Rafie K, Stanley M, Mabbitt PD *et al*: Activity-based E3 ligase profiling uncovers an E3 ligase with esterification activity. Nature. 2018; 556(7701):381-85.

25. Han S, Witt RM, Santos TM, Polizzano C, Sabatini BL, Ramesh V: Pam (Protein associated with Myc) functions as an E3 ubiquitin ligase and regulates TSC/mTOR signaling. Cell Signal. 2008; 20(6):1084-91.

26. Bento CF, Ashkenazi A, Jimenez-Sanchez M, Rubinsztein DC: The Parkinson's disease-associated genes ATP13A2 and SYT11 regulate autophagy via a common pathway. Nat Commun. 2016; 7:11803.

27. Champliaud MF, Baden HP, Koch M, Jin W, Burgeson RE, Viel A: Gene characterization of sciellin (SCEL) and protein localization in vertebrate epithelia displaying barrier properties. Genomics. 2000; 70(2):264-8.

28. van der Vaart B, Manatschal C, Grigoriev I, Olieric V, Gouveia SM, Bjelic S *et al*: SLAIN2 links microtubule plus end-tracking proteins and controls microtubule growth in interphase. J Cell Biol. 2011; 193(6):1083-99.

29. Webb ML, Chao CC, Rizzo M, Shapiro RA, Neubauer M, Liu EC *et al*: Cloning and expression of an endothelin receptor subtype B from human prostate that mediates contraction. Mol Pharmacol. 1995; 47(4):730-7.

30. Martinez-Lopez JE, Moreno-Bravo JA, Madrigal MP, Martinez S, Puelles E: Red nucleus and rubrospinal tract disorganization in the absence of Pou4f1. Front Neuroanat. 2015; 9:8.

31. Wang LX, Wan C, Dong ZB, Wang BH, Liu HY, Li Y: Integrative Analysis of Long Noncoding RNA (lncRNA), microRNA (miRNA) and mRNA Expression and Construction of a Competing Endogenous RNA (ceRNA) Network in Metastatic Melanoma. Med Sci Monit. 2019; 25:2896-907.

32. Chen F, Lukat P, Iqbal AA, Saile K, Kaever V, van den Heuvel J *et al*: Crystal structure of cis-aconitate decarboxylase reveals the impact of naturally occurring human mutations on itaconate synthesis. Proc Natl Acad Sci U S A. 2019; 116(41):20644-54.

33. Asahchop EL, Akinwumi SM, Branton WG, Fujiwara E, Gill MJ, Power C: Plasma microRNA profiling predicts HIV-associated neurocognitive disorder. AIDS. 2016; 30(13):2021-31.

34. Mosca A, Sperduti S, Pop V, Ciavardelli D, Granzotto A, Punzi M *et al*: Influence of APOE and RNF219 on Behavioral and Cognitive Features of Female Patients Affected by Mild Cognitive Impairment or Alzheimer's Disease. Front Aging Neurosci. 2018; 10:92.
