# Supplementary material for: A novel pathogenic frameshift variant unmasked by a large de novo deletion at 13q21.33-q31.1 in a Chinese patient with neuronal ceroid lipofuscinosis type 5
Source: BMC Med Genet. 2020 May 11;21:100. doi: 10.1186/s12881-020-01039-5 (PMC7216669; doi:10.1186/s12881-020-01039-5)
Supplement: Supplementary file 2 — Additional file 2 : Figure S1. The age of onset of CLN5-related features based on published cases. These symptoms include visual failure, motor impairment, walking ability loss, intellectual disability, language impairment, seizure, ataxia. The X-axis shows the age at symptom onset. Red arrows indicate the age of onset of our patient for the respective features. [file 12881_2020_1039_MOESM2_ESM.docx]

**Additional file 2**


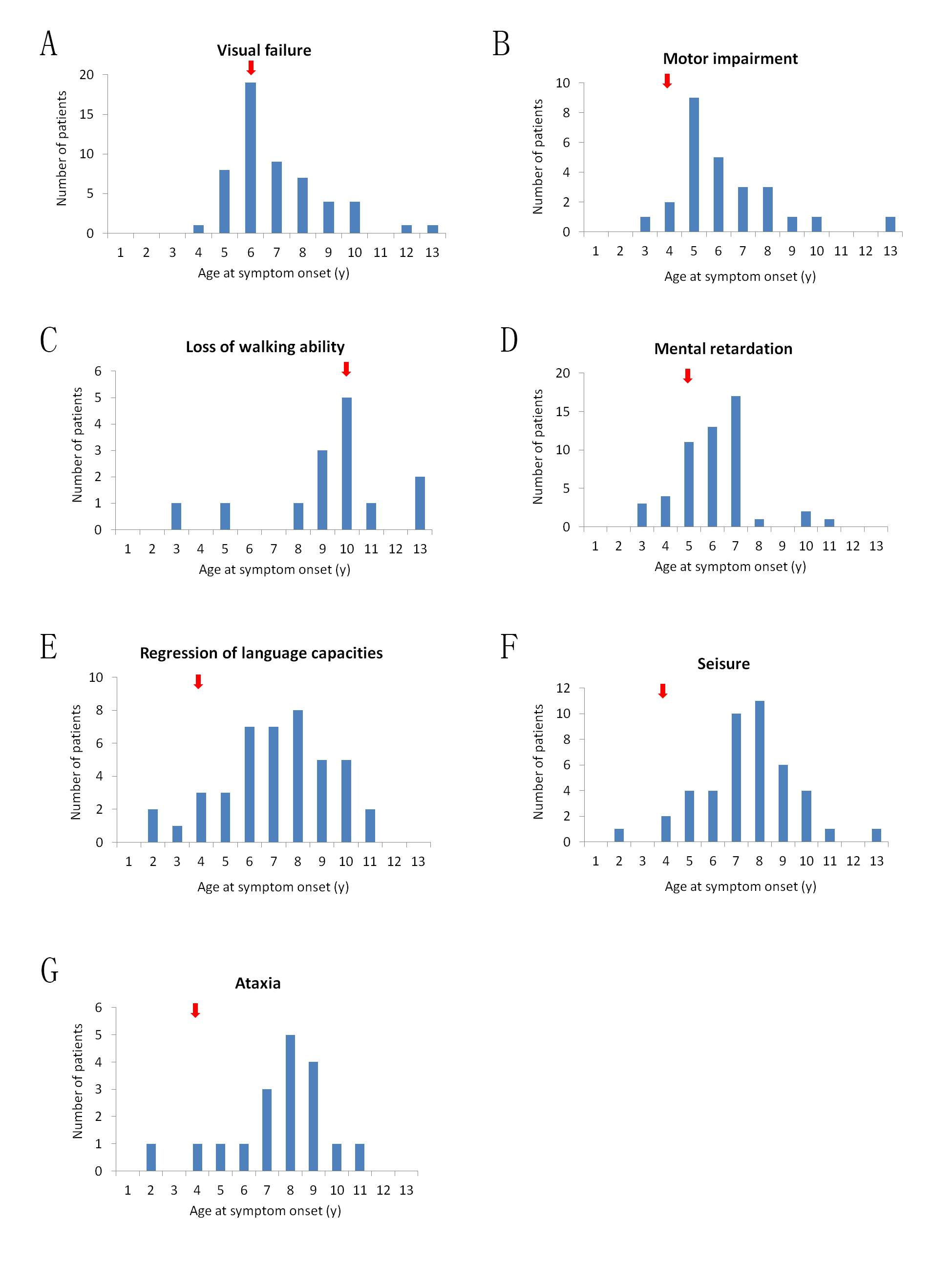


**Figure S1.** The age of onset of CLN5-related features based on published cases. The X-axis shows the age at symptom onset. **a**  Onset ages of visual failure. **b** Onset ages of motor impairment. **c**  Onset ages of walking ability loss. **d** Onset ages of intellectual disability. **e** Onset ages of language impairment. **f**  Onset ages of seizure. **g** Onset ages of ataxia. Red arrows indicate the age of onset of our patient for the respective features.
